# Comparison of Secular Trends in Road Injury Mortality in China and the United States: An Age-Period-Cohort Analysis

**DOI:** 10.3390/ijerph15112508

**Published:** 2018-11-09

**Authors:** Lu Wang, Chuanhua Yu, Ganshen Zhang, Yunquan Zhang, Lisha Luo

**Affiliations:** 1 Department of Preventive Medicine, School of Health Sciences, Wuhan University, 185 Donghu Road, Wuhan 430071, China; ffdw03@whu.edu.cn (L.W.); gszhang1991@163.com (G.Z.); Yun-quanZhang@whu.edu.cn (Y.Z.); 13006362970@163.com (L.L.); 2 Global Health Institute, Wuhan University, 8 Donghunan Road, Wuchang District, Wuhan 430072, China

**Keywords:** age-period-cohort model, road injuries, mortality, trends

## Abstract

This study aimed to identify and compare the mortality trends for road injuries in China and the United States, and evaluate the contributions of age, period, and cohort effects to the trends from 1990 to 2014. Using the 2016 Global Burden of Disease Study database, the mortality trends were analyzed by joinpoint regression and age-period-cohort modeling. Overall, the mortality for road injuries was higher in China than in the United States. The mortality in China increased from 1992 to 2002 (annual percent change [APC] was 1.9%), and then decreased from 2002 to 2015 (APC_2002–2009_ was 1.5%; APC_2009–2015_ was 3.5%). For the United States, the mortality decreased from 1990 to 2010 (APC_1990–1997_ was 1.8%; APC_1997–2005_ was 0.7%; APC_2005–2010_ was 4.2%). Age-period-cohort modeling revealed significant period and cohort effects. Compared with the period 2002–2004, the period risk ratios (RRs) in 2010–2014 period declined by 14.62% for China and 18.86% for the United States. Compared with the 1955–1959 birth cohort, the cohort RRs for China and the United States in the 2010–2014 cohort reduced by 47.60% and 75.94%, respectively. Period and cohort effects could not be ignored for reducing road injury mortalities.

## 1. Introduction

As one of the leading preventable causes of deaths, road injuries are currently estimated to be the ninth leading cause of death across all age groups globally, and are predicted to become the seventh leading cause of death by 2030 [1]. This rise is driven by emerging economies where urbanization and motorization accompany rapid economic growth [2]. Although a decreasing trend in road injury mortality associated with the implementation of various road laws and preventive measures has been observed in a number of developed and developing countries over the last several decades, it remains an important cause of high disability and mortality that threatens the health and lives worldwide [3]. Therefore, in order to better understand the factors that are associated with road injuries trends and assess the effects of public health control policies, an analysis of the secular trends of road injury mortality and their association with the independent effects of age, period, and birth cohort seemed particularly important.

It has been estimated that approximately 1.34 million people died of road injuries in 2016 and more than 1.25 million people die from road injuries each year worldwide [4]. Out of all the fatalities of road injuries worldwide, nearly 90% occurred in low and middle-income countries [5]. As a middle-income country with the number of motor vehicles and transportation volume in increasing substantially in recent years, road traffic injuries have increased prominently in China. On the contrary, although the number of motor vehicles in the United States continues to increase, the number of traffic accidents and the absolute number of deaths have not increased. In 2016, the United States reported approximately 166,174 road injury deaths, while China had nearly 311,605 deaths, which was twice as many as the United States, and ranked first in the world for road injury deaths [6]. Due to the widespread implementation of road safety legislation since its introduction, road injury mortality rates have declined in the United States and China [7,8,9]. However, declines in the United States are steady, and the mortality rate in China remains higher compared with the global level. Overall, the specific reasons for the aforementioned trends in road injury mortality rates remain unclear, and there is an urgent need to conduct more research in order to explore the underlying causes of these trends. By comparing with developed countries, we could find the deficiencies in the road infrastructure, management, or laws in China, and take the reference of American advanced experience for reducing road injuries in China.

The age-period-cohort model (APC) is a popular tool in demography and epidemiology to identify secular trends in disease incidence and mortality rate [10]. The APC model decomposes the mortality rate from the three dimensions of age, period, and cohort, and analyzes the impact of the three factors on the mortality rate simultaneously [11]. As far as we know, current research studies on road injury mortality have focused on short-term trends, or studies limited to specific regions or outdated people. In addition, studies on the comparison of road injury mortality between China and United states using the APC model have been rarely conducted.

Therefore, this study aims to describe and investigate the long-term trends of road injury mortality from 1990 to 2014 in China and the United States, and assess the independent effects of chronological age, period, and birth cohort using the APC model. This study will suggest methods for enhancing the understanding of mortality trends and provide baseline information for the implementation of the SDG (Sustainable Development Goals) target to halve road deaths by 2030.

## 2. Materials and Methods

### 2.1. Data Source

Data were obtained from the Global Burden of Disease Study (GBD) 2016. The GBD study is estimated annually, and each round of results are internally consistent and collectively exhaustive. The GBD 2016 study provided a comprehensive assessment of cause-specific mortality for 264 causes in 195 locations from 1990 to 2016 [4]. Original data, which the GBD adapted to estimate the mortality of road injury, was mainly from vital registration, verbal autopsy, survey/census, and police records. Road injuries were defined with the International Classification of Diseases (ICD-10): pedestrian road injuries (V01–V04.9, V06–V09.9), cyclist road injuries (V10–V19.9), motorcyclist road injuries (V20–V29.9), motor vehicle road injuries (V30–V79.9, V87.2–V87.3), and other road injuries (V80–V80.9, V82–V82.9) [6].

### 2.2. Statistical Analysis

The joinpoint model takes the trend data of mortality rate, and estimates a variation for each point by using a Poisson model of variation. The tests of significance use a Monte Carlo Permutation method [12]. In this study, we used the annual percent change (APC), average annual percent change (AAPC, calculated as geometrically weighted APCs from 1990–2015) and corresponding 95% confidence intervals (CIs) to estimate the age-standardized mortality rates (ASMRs) of road injuries from 1990 to 2015 by the Joinpoint Regression Program Version 4.5.0.

As we know, the collinear interaction effects created by the linear dependency of age, period, and cohort (cohort = period-age) presents a major challenge in long-term trend study. The APC model was fitted to the mortality rate using the age-period-cohort analysis tool provided by the National Cancer Institute online [13]. The model provides parameters that characterize the effects of age, period (year of diagnosis), and cohort (year of birth) related to the observed variations in mortality over time [14]. Since the APC model requires age groups and calendar periods with equal intervals, we divided mortality data into 19 five-year age groups (0–4, 5–9, …, 85–89, 90–94), and five five-year calendar periods (1990–1994, 1995–1999, …, 2010–2014), spanning 23 partially overlapping five-year birth cohorts (1900–1904, 1905–1909, …, 2010–2014).

The longitudinal age curve indicates the fitted longitudinal age-specific rates in the reference cohort adjusted for period deviations [15]. The period Risk Ratio (RR) indicates the ratio of the age-specific rate in each period relative to the reference period. The cohort RR indicates the ratio of the age-specific rate in each cohort relative to the reference cohort [13]. In this study, the reference age group was 45–49 years, and the reference period was 2000–2004, which determined that the reference cohort was 1955–1959. The net drift and local drifts are important parameters in the APC model. The net drift is analogous to the overall annual percentage change adjusted for age group over time, and the local drifts are expressed as an estimated annual percentage change value for each age group [16]. Wald Chi-Square tests were adopted for the significance of the estimable parameters and functions, and *p*-values less than 0.05 were often considered statistically significant [17].

## 3. Results

### 3.1. The Overall Trends of Mortality Rate from Road Injuries

**Figure 1:**
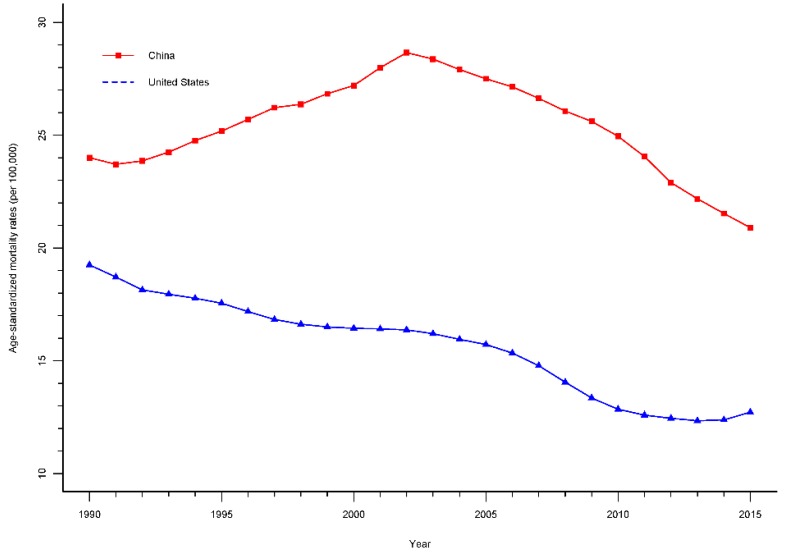
The trends of age-standardized mortality rates for road injuries in China and the United States, 1990–2015.

The trend of ASMRs per 100,000 population for road injuries in China and the United States from 1990 to 2015 are shown in Figure 1. The total ASMRs from road injuries in China declined by 13.2% from 1990 to 2015, showing a tendency of increase first and then decrease, while for the United States, the ASMRs was lower than in China, and consecutively decreased by 33.5% from 19.3% in 1990 and 12.7% in 2015.

As shown in Table 1, the ASMRs for road injuries in China decreased slowly by 0.3% (95% CI: −1.8% to 1.3%) per year from 1990 to 1992, and then increased 1.9% (95% CI: 1.7% to 2.0%) per year from 1992 to 2002, and decreased 1.5% (95% CI: 1.7% to 1.2%) per year from 2002 to 2009 and 3.5% (95% CI: 3.8% to 3.3%) per year from 2009 to 2015. For the United States, the ASMRs showed a consecutive significant decrease from 1990 to 2010 (APC_1990–1997_ was 1.8%, 95% CI: 2.1% to 1.5%; APC_1997–2005_ was 0.7%, 95% CI: 1.0% to 0.4%; APC_2005–2010_ was 4.2%, 95% CI: 4.8% to 3.6%), then a decrease of 1.5% (95% CI: −3.6% to 0.6%) per year from 2010 to 2013, and an increase of 1.6% (95% CI: −0.5% to 3.8%) per year from 2013 to 2015.

### 3.2. The Age, Period, Cohort Effects of Mortality Rate from Road Injuries

**Figure 2:**
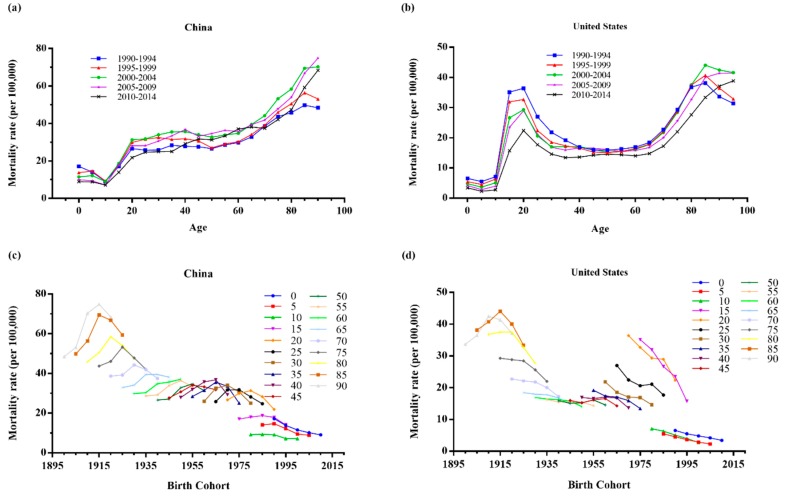
Age-specific mortality rates of road injuries by periods and cohort-specific mortality rates of road injuries by age groups in China and the United States. (**a**,**b**) represented the age-specific mortality; (**c**,**d**) represent the cohort-specific mortality.

Figure 2a,b indicated the existence of a period effect of the road injury mortality in China and the United States. The mortality rates for two countries under 15 years old were lower, showing a declining trend. Regardless of the period effects, the mortality rates in China continued to rise over 15 years old, plateaued from 20 to 49 years, and then increased appreciably after 50 years. The age-specific mortality rates fluctuated unpredictably with the period changes. However, the age-specific mortality rates from road injuries by periods in the United States over 15 years old presented a U-shaped pattern with two peaks in the age groups of 15–24 and 85–89 years old. It is remarkable that the mortality rates in the same age group presented a declining trend from the period 1990–1994 to 2010–2014, and then demonstrated an irregular fluctuating trend for the age group over 80 years old.

Figure 2c,d suggested the existence of a cohort effect of mortality from road injuries in China and the United States. With the increase of age, the mortality rates from road injuries in China presented a decreased trend from the birth cohort 1900–1904 to 2010–2014. Within the same age group, the mortality increased firstly and then decreased. Generally, the older age groups suffered from a higher risk of road injury mortality than the younger age groups, especially in the age groups 80–84, 85–89, and 90–94. In addition, the birth cohort mortality rate under 15 years old experienced the lowest mortality rate and tended to decline.

With the increase of birth cohorts, the mortality rate in the United States demonstrated a U-shaped pattern, except for the age group 0–14, and the lowest occurred in the birth cohort 1940–1944. Within the same age group, the mortality rate demonstrated an uneven decrease, with the steepest decrease in the age groups 15–19 and 20–24 years old, except for the age groups over 85 years.

**Figure 3:**
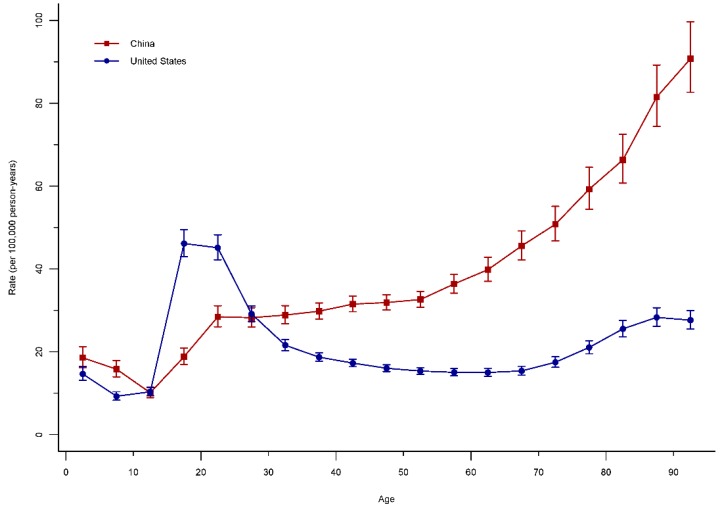
Longitudinal age curve of road injury mortality rate under the APC framework.

The longitudinal age curves of road injuries for China and the United States were displayed in Figure 3. Generally speaking, the mortality rate of road injuries in China continued on an upward trend for all age groups, except for the age group from 0 to 9 years old. It was noted that the fastest-growing age group was 15–19 years. In the United States, the mortality rate of road injuries adjusted for period deviations witnessed a slight decline in the under 5–9 age groups, and then a substantial increase from the age group of 15–24 years that was even higher than the increase for the same groups in China. Using 20–24 years as the reference age group, the mortality in China kept continuously rising, while the United States displayed a downward pattern. Although the mortality in the United States rebounded marginally since the age of 65, it was still well below that of China.

**Figure 4:**
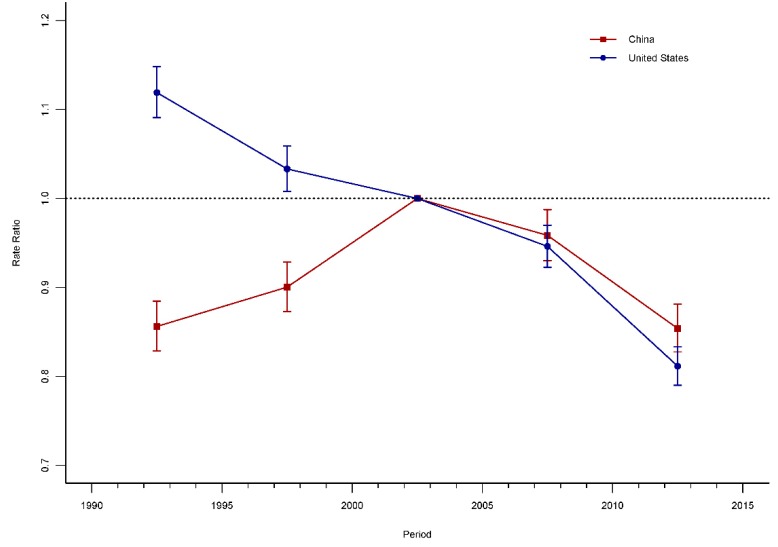
Period RRs of road-injury mortality rate adjusted for age and birth cohort effects, compared to the referent period (2000–2004) and the corresponding 95% CI.

**Figure 5:**
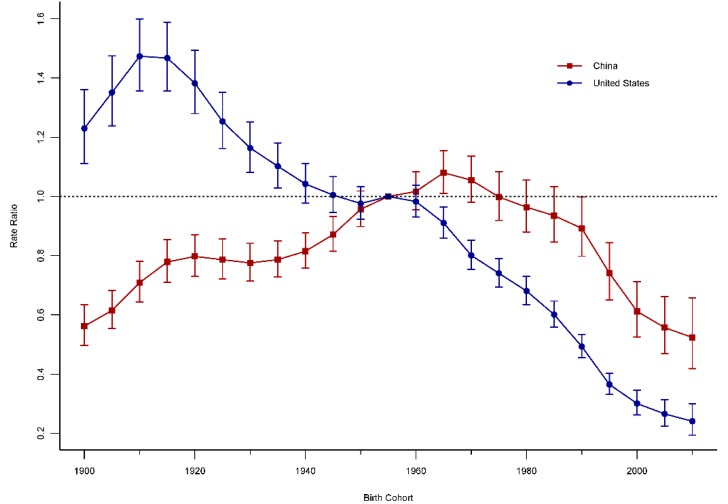
Cohort RRs of road injury mortality rate adjusted for age and period effects, comparing to the referent cohort (1955–1959) and the corresponding 95% CI.

The age-period-cohort modeling indicated the existence of the period and cohort effects in the mortality trends of road injuries for China and the United States, with period and cohort deviations significantly different from zero (*p* < 0.001 for all, Wald tests). The period effects of road injuries for China and the United States are displayed in Figure 4. The period RRs for road injuries in China increased firstly and then decreased, while the United States presented monotonic downward patterns. Compared with 2002–2004, the period RR for road injuries in the period 2010–2014 declined approximately 15% (14.62% for China and 18.86% for the United States). The cohort of road injuries for China and the United States are displayed in Figure 5. The cohort RRs displayed a similar trend with the period RRs for the two countries. Compared with the birth cohort 1955–1959, the cohort RR for China and the United States in the cohort 2010–2014 were reduced by 47.60% and 75.94%, respectively.

**Figure 6:**
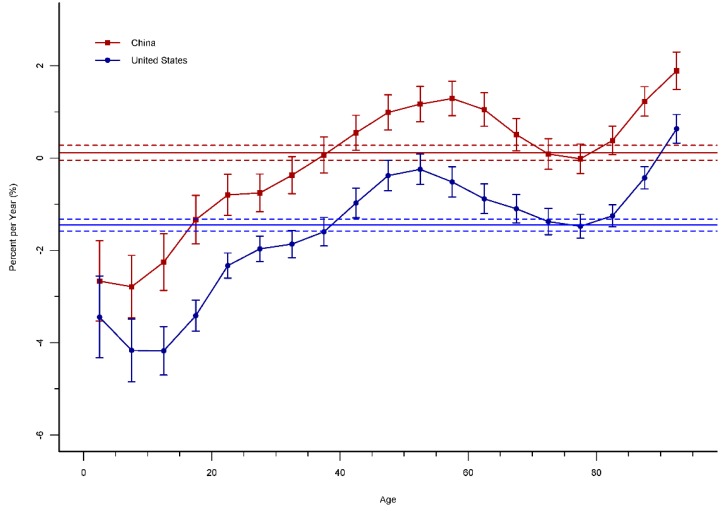
Local drift and net drift with 95% confidence intervals in road injury mortality for China and the United States.

The local drift values are an estimated annual percentage change value for each age group, and are presented in Figure 6. The local drift values in China were below 0 in most of the age groups except for 35–74 years and over 80 years, and were lowest in the age group 5–9 years (around −2.79% per year). In the United States, the local drifts were below 0 in almost all of the age groups except for the age group 90–94, which were lowest in the age group 10–14 years (around −4.18% per year). In addition, the most of local drifts in China were higher than those in the United States. As references, the net drift values, which indicate the overall APC within the observed period, were −0.11 (95% CI: −0.05 to 0.27) for China and −1.45 (95% CI: −1.58 to −0.32) for the United States, respectively.

In addition, the results of the Wald tests demonstrated that there were statistically significant cohort and period RRs for both countries (*p* < 0.05 for all, except the net drift for China), and so were the net drift and local drift (*p* < 0.05 for all). Besides, the period deviations were far less than the cohort deviations for the two countries, which indicated that the period RRs with road injury mortality were mainly reflected by the net drift.

## 4. Discussion

This study assessed the mortality trends of road injuries for China and the United States, and examined the independent effects of age, period, and birth cohort under the APC framework. Our results demonstrated that the mortality of road injuries displayed different trends for the two countries, and further suggested the existence of period and cohort effects in road injury mortality.

Although road injuries may occur to people of all ages, age is still one of the most important risk factors for deaths. Using the latest data from GBD 2016, our results revealed that the risk of death from road injuries in the same birth cohort had a rapid rise at the age of 15–24 years and were both extremely higher among old age groups after adjusting for period and cohort deviations. The former is mainly due to the characteristics of young people, the types of vehicles they used, their education, the cognition differences regarding cars, etc. As we know, the legal drinking age in the United States is 21 years old, and the age for obtaining a driving learner’s permit is 14–16 years old. In China, the minimum age for a driver’s license is 18 years old. Young people in these age groups are inexperienced, immature, adventurous, and exposed to greater risks [18,19]. A large proportion of teenagers leave compulsory school, began to pursue stimulation, such as smoking, drinking, speeding, etc. Inadequate driving skills and drunk driving accelerate the road injury mortality rate rapidly [20]. The types of vehicle used at this age are more likely to be motorcyclists for male and cyclists for female [3]. Moreover, as the vulnerable road users, motorcyclists and cyclists are the most frequent victims of traffic fatalities. Research has shown that road injuries are the second leading cause of death for 15–24 year-olds in the United States [21]. The differences in education and people’s cognition about cars between China and the United States has resulted in more people aged 15–24 years driving in the United States, and a higher mortality rate of road injuries. At the age of 15–24 years, Chinese citizens are mainly studying in school. Due to tying the cars to their wealth or social status, they are more willing to buy new cars once they can afford them. In contrast, American aged 15–24 years enjoy more freedom and autonomy. Using cars as a tool, many people are used to renting a car or buying a used car. In addition, the phenomenon where the mortality rate in the United States at the age of 15–24 years is higher than that in China may be related to the ethnic differences [22]. Furthermore, our results were consistent with the internationally recognized awareness of the high-risk age groups of death [23,24]. The elderly people had the highest mortality rate of road injuries for both countries. This is mainly because the elderly are very vulnerable in the road environment, whether they are drivers or pedestrians. Whether in response to traffic conditions or after a collision, they may experience more serious damage or recover more poorly due to exposure and vulnerability [25]. Studies have shown that the travel mileage of the elderly is about five times that of young people. The driver in fatal crashes for the oldest age group is over 30% higher than the next oldest group [26].

The period effects referred to the risk change for all of the age groups due to environmental changes and changes in diagnostic criteria and disease classification [14,27]. Compared with 2000–2004, the risk of road injuries in the United States between 1990–1994 and 1995–1999 were higher, and showed a downward trend. In contrast, the mortality in China was lower and constantly increased. Then, the risk of road injuries in China since 2000–2004 was higher than that in the United States, and both kept a downward trend.

We think there are many reasons for this result, such as the implement of road laws and preventive measures, the development of road safety-enhancing infrastructure, the improvement of regulation, etc.

Both countries have enacted many different road laws and preventive measures during the period 1900–2014. Since 1996, China has gradually promulgated traffic laws and preventive measures, which led to a reduction in the mortality of road injuries. In 2004, the National People’s Congress passed comprehensive road traffic safety legislation [28]. Although the laws in 2004 had initially formulated various aspects of road safety, the regulations on drunk driving, seat belts, speed control, etc. were still not specific, and implementation was not enough. Later, China revised the road safety laws in 2011, which made drunk driving a criminal offense and introduced severe penalties for drunk driving, which made people pay more attention to drunk driving [29]. So, the mortality rate of road injuries began to decline after 2005. Likewise, as early as the 1960s, the United States gradually enacted traffic regulations to reduce the high mortality caused by road injuries. The State of New York passed legislation requiring drivers and passengers in motor vehicles to wear seat belts in 1984. This is the first state legislation to enforce seat belts in the United States. At the beginning of this century, the United States made corresponding regulations and penalties for distracted driving, while China began to pay close attention to it in 2013. At present, the United States has not only the world’s largest fleet of motor vehicles, but also a well-developed road traffic management system.

The preventive measures have greatly reduced the mortality of road injuries. Alcohol abuse is a well-known cause of road injuries. Drunk driving not only could increase the risk of self-driving, but also increase the risk of severe injury to other road users by a factor of four [30]. There is overwhelming evidence that the strict enforcement of drunk-driving policies could result in a significant reduction in alcohol-related road injuries [31]. The seat belt, the most important measure of road injury safety, could reduce the severity of the injury, the length of hospital stay, and the number of operations in the injured patient [32,33]. The older drivers without seat belts were five times more likely to be injured than younger people. The use of seat belts reduced the severity of injuries to the elderly the most [34]. Studies also revealed that there was a significant drop in certain types of injuries due to road injuries after the enactment of the seat belt law [5,35].

It is well-known that the road is the main infrastructure of the transportation industry. In the early 20th century, road construction and planning were not synchronized with the development of highway transportation, which led to higher risks of road injuries, especially in the United States. The United States is sparsely populated, and people’s work and life are very dependent on vehicles. During the same period, road development in China had not kept up with economic development, and the constraints did not end until the 21st century. People in China only used the highway when traveling or transporting. So, between 1990–1994 and 1995–1999, the risk of road injuries in the United States was higher than in China, and displayed the opposite trend. While the United States built the highway network in the 1980s, China formed the main road network of the expressway in the decade after 2000. The roads in the United States are wide and plain, and maintenance is simple and efficient. However, that road construction in China started late with high standards made road maintenance difficult. Maintenance makes roads narrow and congested, which increases the chance of traffic accidents. So, the risk of road injuries after 2000–2004 for the two countries both declined, but the risk in China was higher than in the United States.

In addition, the management of the road transportation was different in the two countries, resulting in a faster decline in the mortality of road injuries in the United States than in China. First, the United States improved the safety and efficiency of transportation by using intelligent traffic management. However, the management of traffic in China involves many departments such as the transportation department, public security department, and tax department, whose support for the transport industry is not uniform. Secondly, the strict judgments on illegal driving such as drunk-driving, illegal racing, and overloading are more stringent, and the punishments are more severe. The above management may are also worth learning for China. The change from the International Statistical Classification of Diseases and Related Health Problems (ICD) 9 and ICD-10 had a substantial influence on the analysis of temporal trends for unintentional injury mortality [36]. Although no research has found that this change has an impact on road injury mortality, it is not without this possibility. Hence, although the mortality of road injuries for the two countries has decreased since 2000–2004, China has suffered from a higher risk than the United States.

The birth cohort effect may be due to uneven population exposure or unequal exposure levels in different age groups who are at a critical development period [27]. Compared with the cohort effects in 1955–1959, the risk of road injuries in the United States between the 1900–1904 cohort and the 1945–1949 cohort were higher, but presented a downward trend; in contrast, the risk in China was lower and kept constantly increasing until the 1965–1969birth cohort. Since the 1965–1969 birth cohort, the risk of road injuries was higher in China than in the United States, and both kept a downward trend.

The cohort effects of road injuries are elaborated by the following aspects: social history, the number of population, the development of the automobile industry and road infrastructure, road safety awareness, medical standards, and so on. Firstly, on the one hand, the “First World War”, the “Spanish Flu”, and the “Second World War”, which caused a large number of deaths in the United States, made the mortality of road injuries higher. On the other hand, the United States in 1920 enacted the “Volstead Act”, which lasted for 14 years and reduced risk of the road injuries caused by the drunk driving. During the 1960s and 1970s, good fertility and increasing immigration in the United States have boosted population growth, which also reduced the road injury mortality. In contrast, the risk of road injuries in China between the 1900–1904 and 1950–1954 cohorts were lower, but presented an upward trend. On one hand, the world wars or natural disasters that resulted in mass migration or deaths made the total population decline, which increased the mortality of road injuries in China. The levels of economic development and heavy industry were relatively backward, which made the risk of road injuries lower. On the other side, the road injury mortality increased with the rapid development of the automobile industry in China during 1949–1999. However, the total population was the fastest growing at the same time, which increased the population base and inhibited the increase in road injury mortality. Secondly, the United States enacted the Federal Aid Road Act in 1916, and began to develop road construction. However, the construction of road infrastructure in China achieved leapfrog development after China’s reform and opening up in 1978. Therefore, the risk of people born in 1915–1919 for death by road injury in the United States began to decrease, while the decline in China occurred in the 1975–1979 birth cohort. Thirdly, traffic safety education has been incorporated into American students’ classes, but in China, only drivers have enforced education in this area. Although China has always stressed that it is necessary to improve the road safety awareness of all people except for the drivers, the safety awareness of others is still weak. In addition, the medical service level also has an important role in the mortality rate of road injuries. As the largest developed economy, the United States’ medicine service is in an advanced position in the world from the scope and depth of application. The United States is ahead of China in the medical system construction, including medical levels and informatization. The other risk factors, such as road types, weather factors, and vehicle types may also influence the road injury mortality.

There were some limitations to this study. Firstly, the major limitation of our study is that the precise quantitative conclusions depend on the accuracy of the GBD 2016 methods. While the GBD 2016 has made many corrections to the source, collation, and evaluation methods of data to improve data quality and comparability, compared to secondary data, we tend to prefer raw data more. Secondly, we only obtained age data in five-year intervals due to the limitation of the data and the APC method, which made us miss more detailed conclusions regarding road injury mortality. Thirdly, in this study, we only estimated the risk factor of age, period, and cohort in road injuries, and made no further analysis of other risk factors.

## 5. Conclusions

In summary, we evaluated the general trends in the mortality rates of road injuries in China and the United States during 1990–2014, and the contribution of age, period, and cohort effects to the trends. Overall, the mortality in China increased firstly and then decreased later, while the United States steadily decreased. Through the age-period-cohort model, the declining trends were observed in the period and cohort effects for the two countries; however, the declining trends in the United States were earlier and faster compared to the trends in China, which may be attributed to the more developed and complete road transportation network in the United States. Additionally, the risk of mortality began to increase rapidly at the 15–24 year age group in China and the United States, and increased continuously in older age groups. Although the age effect was relatively strong, the period and cohort effects may be the key factors affecting trends in road injury mortality, mainly reflecting the importance of improving the road infrastructure, strictly implementing the road laws and preventive measures, employing intelligent traffic management, and raising the awareness of road safety for all.

## Figures and Tables

**Table 1:** Annual percent change in road injury mortality rates for China and the United States, 1990–2015.

Segments	China		United States	
	Year	APC (95% CI)	Year	APC (95% CI)
**Trend 1**	1990–1997	−1.8 (−2.1, −1.5) *	1990–1992	−0.3 (−1.8, 1.3)
**Trend 2**	1997–2005	−0.7 (−1.0, −0.4) *	1992–2002	1.9 (1.7, 2.0) *
**Trend 3**	2005–2010	−4.2 (−4.8, −3.6) *	2002–2009	−1.5 (−1.7, −1.2) *
**Trend 4**	2010–2013	−1.5 (−3.6, 0.6)	2009–2015	−3.5 (−3.8, −3.3) *
**Trend 5**	2013–2015	1.6 (−0.5, 3.8)		
**AAPC (95% CI)**	1990–2015	−0.6 (−0.7, −0.4) *	1990–2015	−1.6 (−1.9, −1.3) *

* The value is statistically different from zero (*p* < 0.05) based on joinpoint regression analysis. AAPC, average annual percent change; APC, annual percent change; CI: confidence interval.
